# Supplementary material for: A Novel Oncogenic Role of FDX1 in Human Melanoma Related to PD-L1 Immune Checkpoint
Source: Int J Mol Sci. 2023 May 24;24(11):9182. doi: 10.3390/ijms24119182 (PMC10253061; doi:10.3390/ijms24119182)
Supplement: Supplementary file 1 [file ijms-24-09182-s001.zip › ijms-2349058-supplementary.pdf]

# *Supplementary Material*

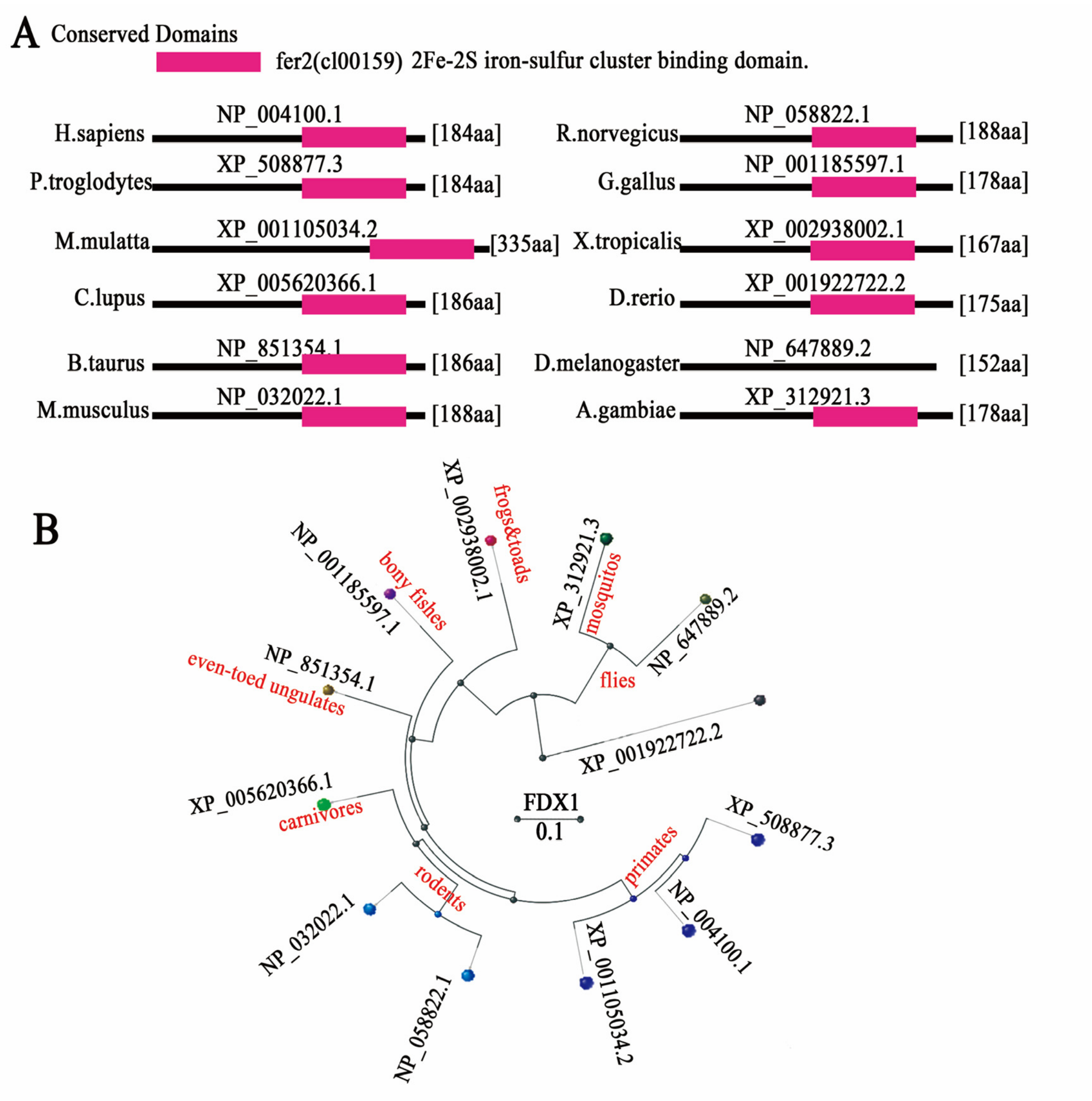


**Figure S1.** the structural features of FDX1 and phylogenetic tree in diverse species (**A**). the conserved domains of FDX1 protein from various species (**B**). We also displayed the phylogenetic tree of FDX1 in diverse species through constraint-based multiple alignment tool of NCBI dataset.

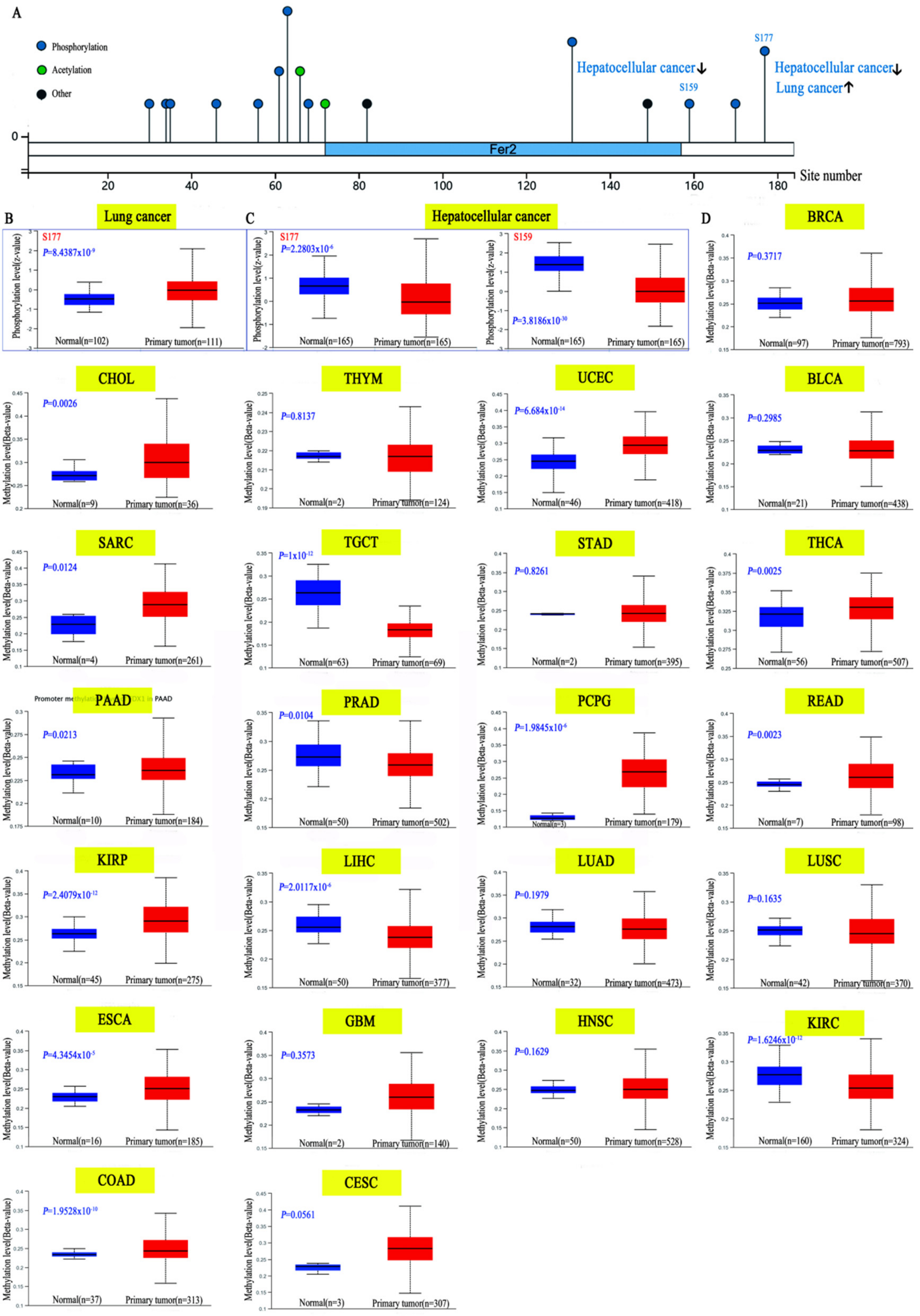


**Figure S2.** Protein phosphorylation and DNA methylation of FDX1 in human multicancers. According to CPTAC tool, we displayed the FDX1 phosphoprotein sites including S177 and S159 between normal and primary tissues in various human tumors, and positive results were shown in the schematic diagram of FDX1 (**A**). Moreover, the box-plots for diverse tumors, such as lung cancer and hepatocellular cancer (**B** and **C**). We also conducted the box-plots of methylated sites in various tumors including BRCA, CHOL, THYM, UCEC, BLCA, SARC, TGCT, STAD, THCA, PAAD, PRAD, PCPG, READ, KIRP, LIHC, LUAD, LUSC, ESCA, GBM, HNSC, KIRC, COAD and CESC (**D**).

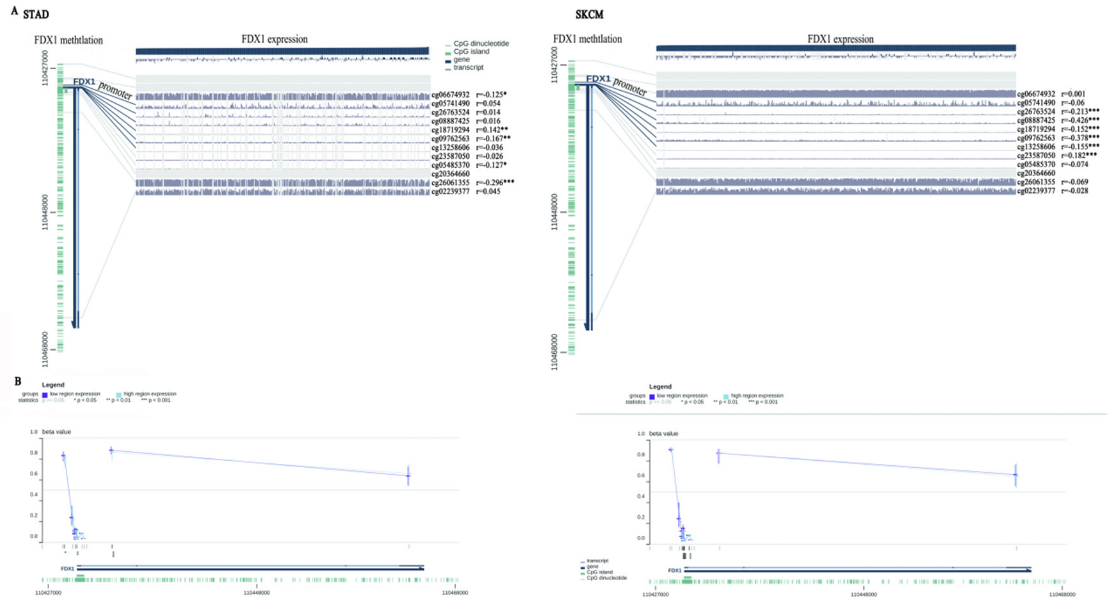


**Figure S3.** The correlation between FDX1 expression and DNA methylation through the MEXPRESS tool of TCGA dataset in (**A**) STAD and in (**B**) SKCM. * $p < 0.05$; ** $p < 0.01$; *** $p < 0.001$.

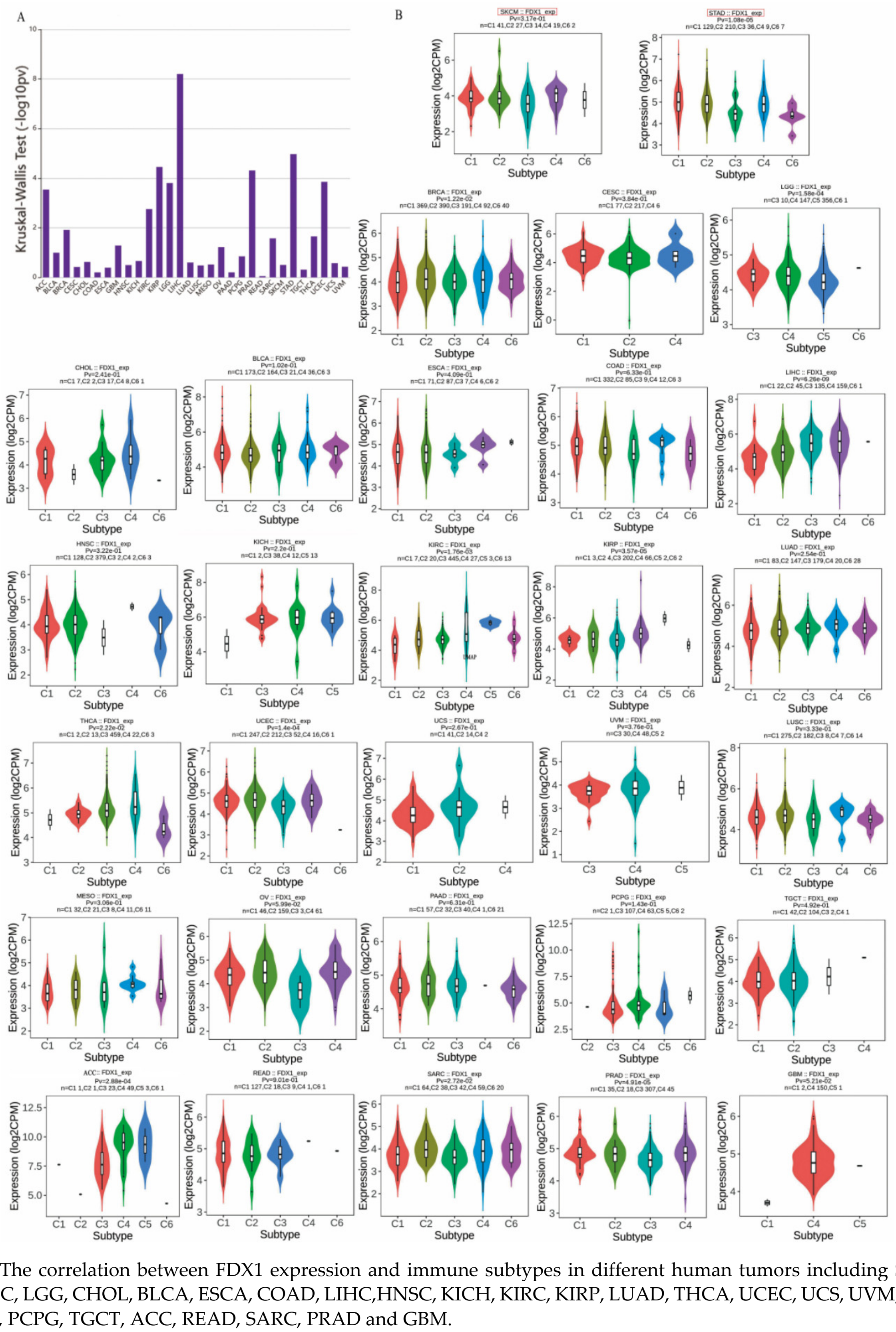

**Figure S4.** The correlation between FDX1 expression and immune subtypes in different human tumors including SKCM, STAD, BRCA, CESC, LGG, CHOL, BLCA, ESCA, COAD, LIHC,HNSC, KICH, KIRC, KIRP, LUAD, THCA, UCEC, UCS, UVM, LUSC,MESO, OV, PAAD, PCPG, TGCT, ACC, READ, SARC, PRAD and GBM.

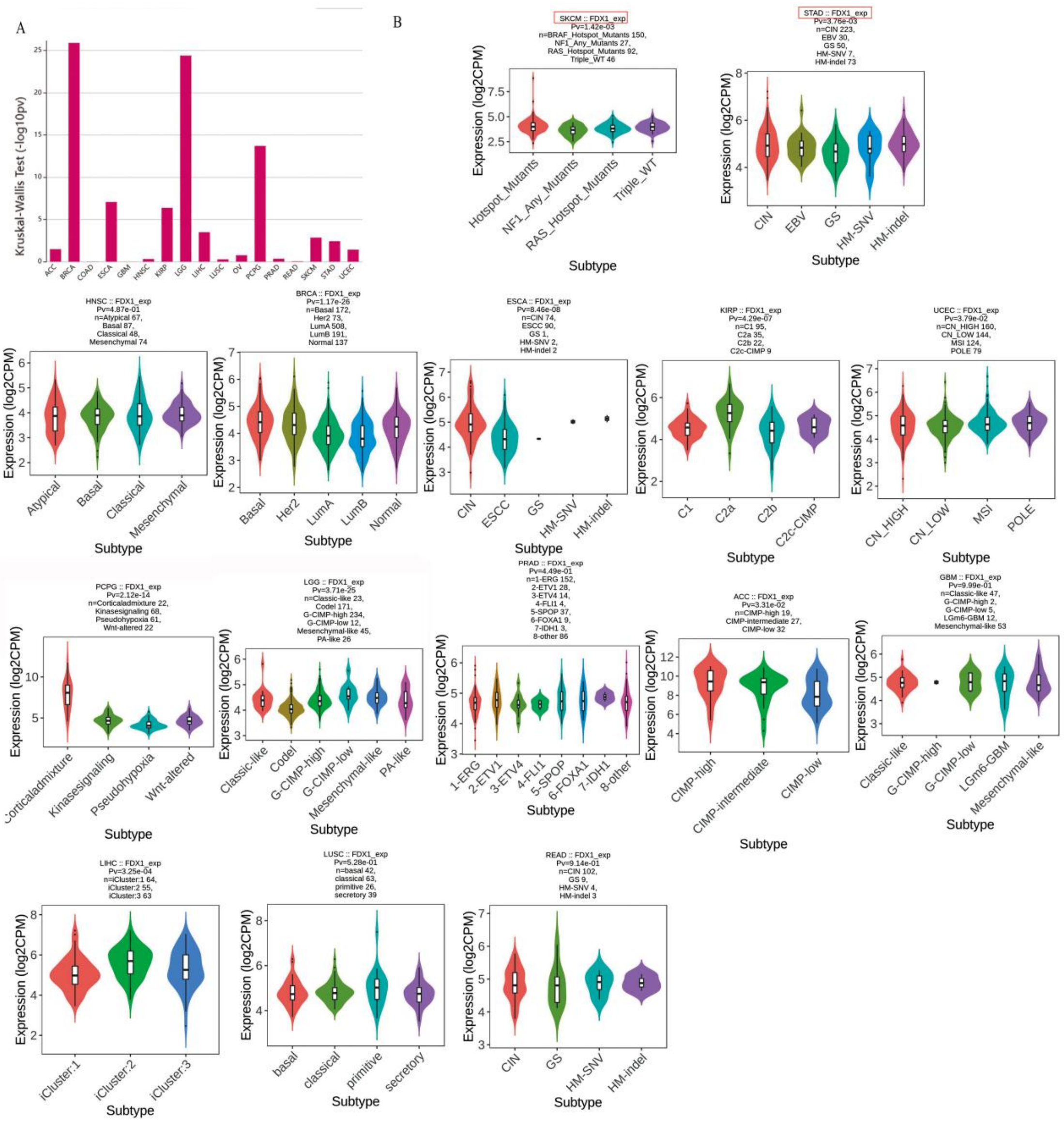

**Figure S5.** The correlation between FDX1 expression and molecular subtypes in different human tumors including SKCM, STAD, HNSC, BRCA, ESCA, KIRP, UCEC, PCPG, LGG, PRAD, ACC, GBM, LIHC, LUSC and READ.

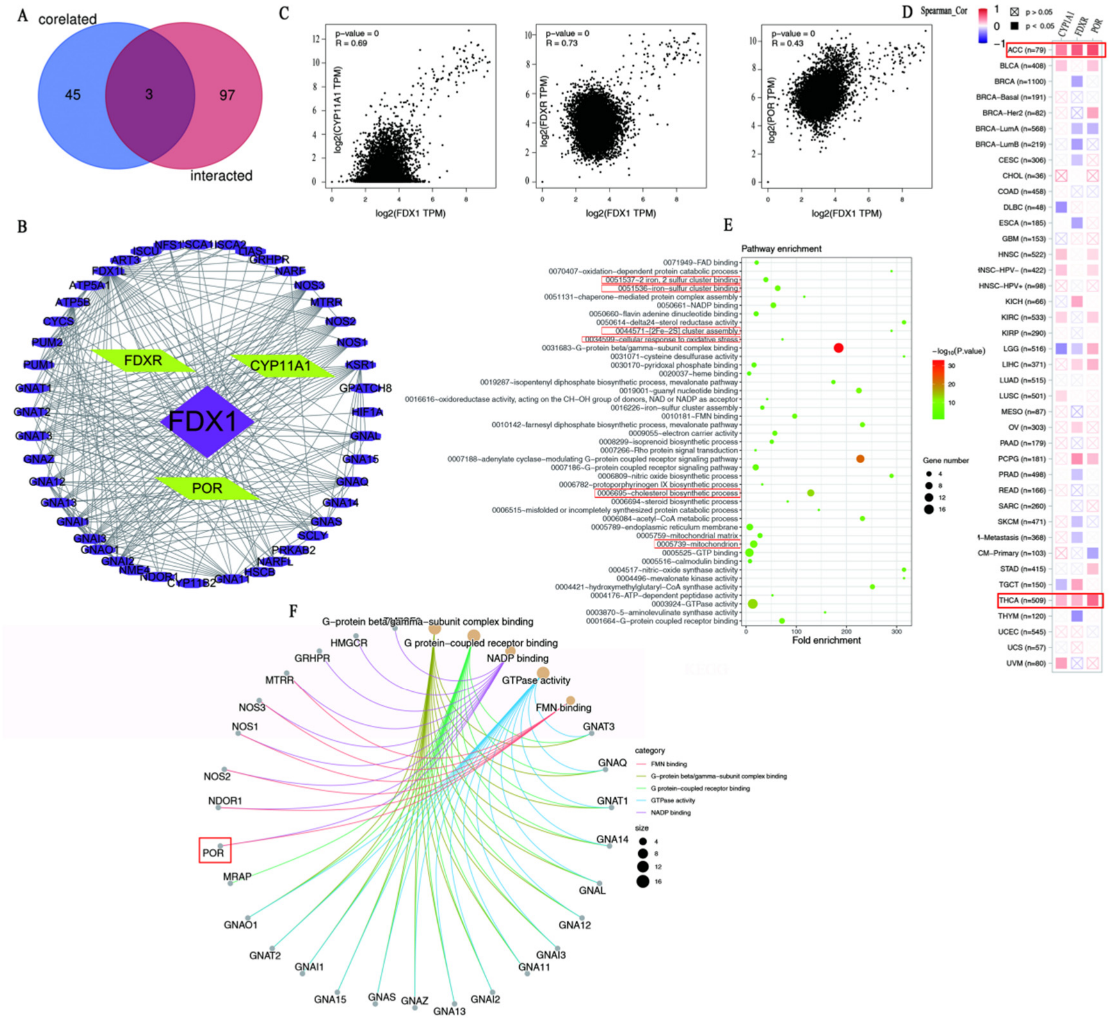


**Figure S6.** The analysis of FDX1-related gene,s GO/KEGG enrichment. An intersection of FDX1-binding genes and the network of FDX1-binding proteins were displayed through Veen and STRING websites. We also found the top-100 FDX1-related genes based on GEPIA2 of TCGA dataset, and gained the closest correlation between FDX1 and three hub-genes, such as FDXR, CYP11A1 and POR (**A** and **B**). The scatter diagram and heat-map of hub-genes are shown in different cancer types (**C** and **D**). We performed the cnetplot of GO/KEGG enrichment through FDX1-binding and interacted genes (**E** and **F**).

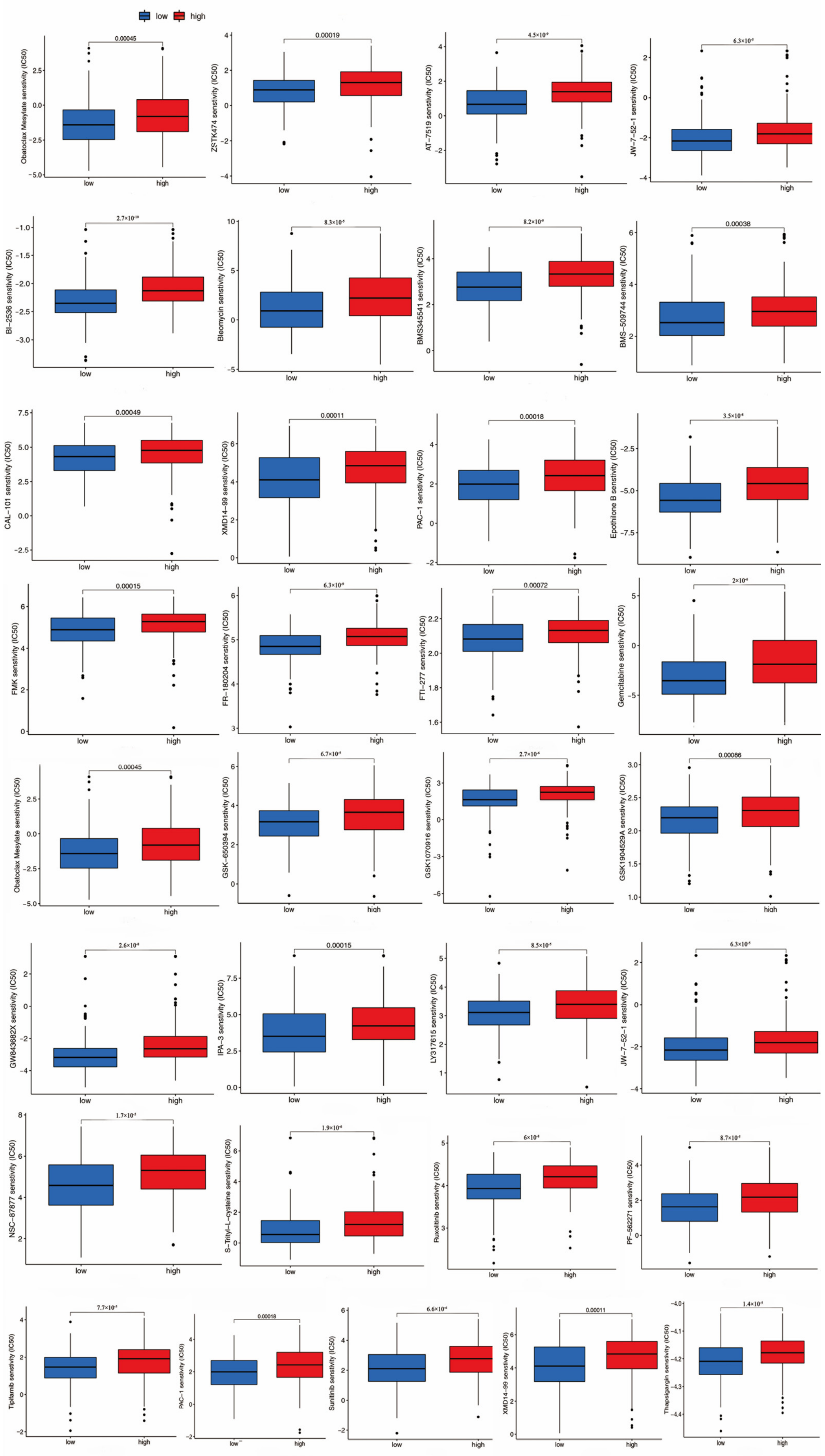


**Figure S7.** The correlation of FDX1 expression and drug sensitivity was shown by Box plots and tested with Pearson correlation from NCL-60 cell line data.

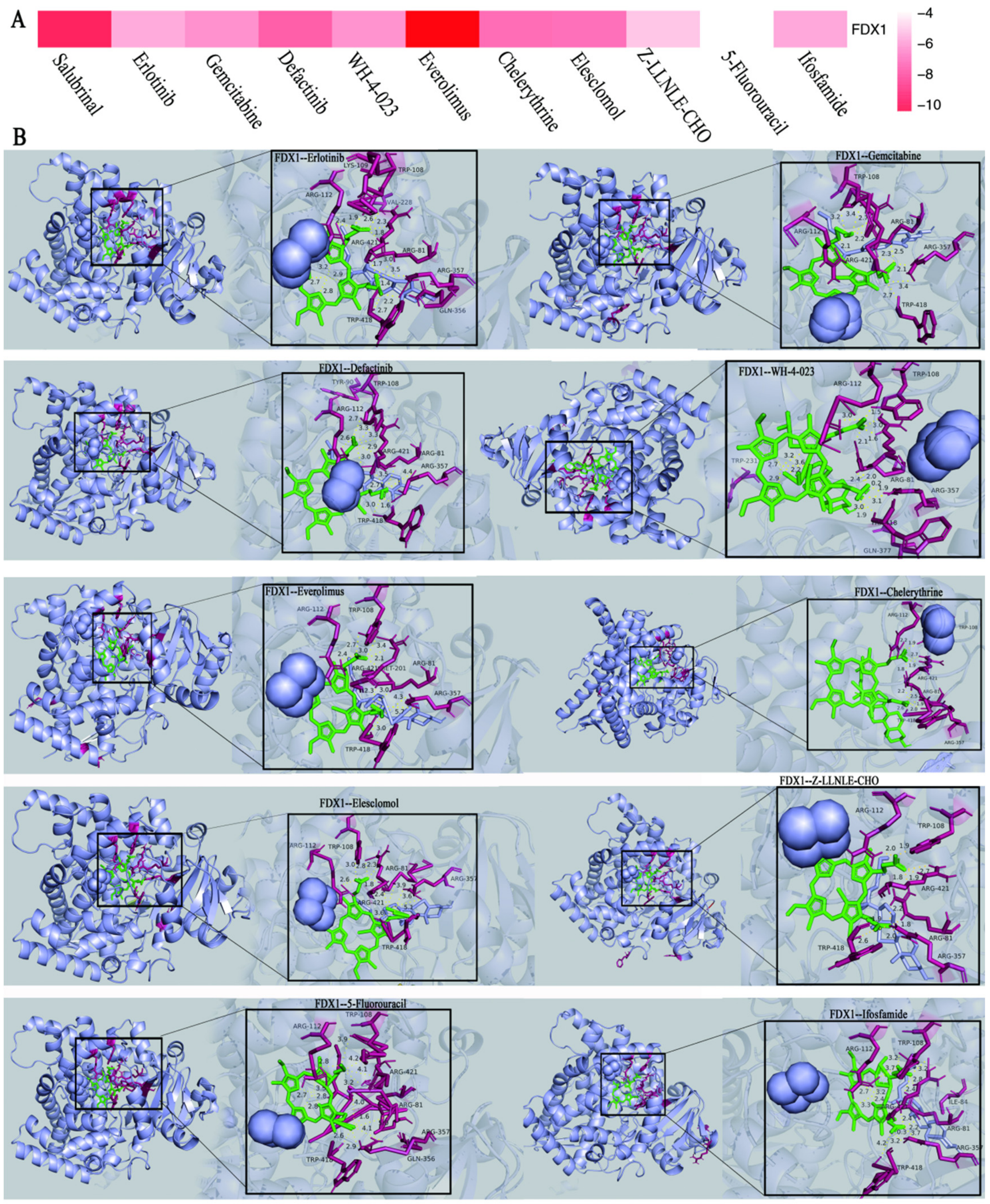


**Figure S8.** The interaction between FDX1 and Salubrinal, Erlotinib, Gemcitabine, Defactinib, WH-4-023, Everolimus, Chelerythrine, Elesclomol, Z-LLNLE-CHO, 5-Fluorouracil, Ifosfamide was shown by bar plots and red presents binding degree (**A**). the total view and detailed view of FDX1(**B**).

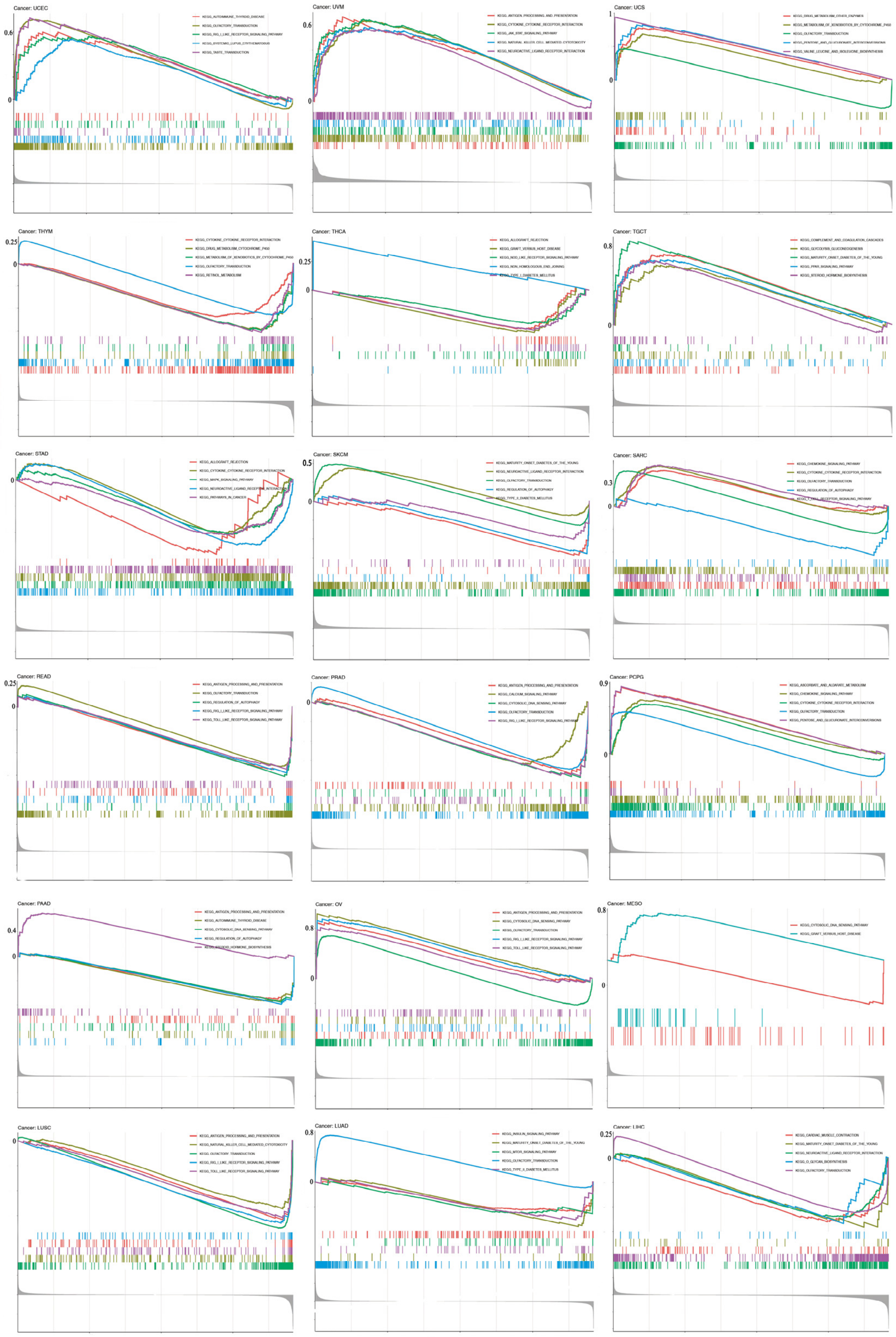

**Figure S9.** The analysis of FDX1 expression and gene set enrichment in different cancer types including UCEC, UVM, UCS, THYM, THCA, TGCT, STAD, SKCM, SARC, READ, PRAD, PCPG, PAAD, OV, MESO, LUSC, LUAD, LIHC, ACC, LGG, LAML, KIRP, KIRC, KICH, HNSC, GBM, ESCA, DLBC, COAD, CHOL, CESC and BLCA. X-axis represents the sequencing of enriched genes, and Y-axis represents the enrichment-score. X-axis represents rank in ordered dataset and Y-axis represents running enrichment score.
